# EEG alpha reactivity in cognitive aging and dementia: clinical implications and cholinergic mechanisms

**DOI:** 10.3389/fnhum.2026.1665301

**Published:** 2026-02-27

**Authors:** Kwangyeol Baek

**Affiliations:** School of Biomedical Convergence Engineering, Pusan National University, Busan, Republic of Korea

**Keywords:** aging, alpha reactivity, alpha rhythm, Alzheimer's disease, dementia, electroencephalography, Lewy body dementia, nucleus basalis of Maynert

## Abstract

Alpha reactivity—the attenuation of the alpha rhythms upon eye-opening—is a well-known phenomenon in electroencephalography (EEG). Altered alpha reactivity has increasingly been recognized as a potential biomarker for cognitive aging and various types of dementia. This mini-review synthesizes the existing literature on EEG alpha reactivity in older adults, highlighting its clinical implications and neurobiological underpinnings. Methodological issues in quantifying alpha reactivity are first addressed, including the choice of reactivity index, frequency bands, and spatial analysis methods. The review then summarizes evidence that alpha reactivity declines with healthy aging and is further reduced in dementia, especially Alzheimer's disease (AD) and Lewy body dementia (LBD). Importantly, distinct patterns of reduced alpha reactivity may aid in differential diagnosis. It has been reported that AD is often characterized by reduced alpha rhythm during eyes-closed rest, whereas LBD typically shows impaired alpha attenuation upon eye-opening. Neuroimaging and pharmacological studies suggest that alpha reactivity reflects cholinergic system integrity, particularly involving the nucleus basalis of Meynert and its projections to the visual cortex. In conclusion, EEG alpha reactivity is a promising, non-invasive biomarker of cognitive health and dementia subtypes. However, larger, harmonized, multimodal and longitudinal studies are needed to establish its diagnostic value and clarify its neurobiological mechanisms.

## 1 Introduction

The posterior-dominant alpha rhythm (PDR), typically ranging from 8–13 Hz, is prominent during eyes-closed rest and is markedly attenuated upon eye opening (Britton et al., 2016; Kane et al., 2017). This well-known phenomenon, originally described by Berger (1929), is often referred to as “alpha reactivity,” “alpha attenuation,” or “alpha blocking” (Partanen et al., 1997; Wan et al., 2019). Alpha reactivity is quantified by relative change of alpha power between eyes-closed (EC) and eyes-open (EO) conditions, while alpha power refers to the absolute or normalized spectral power of EEG rhythm within the alpha frequency range (typically 8–13 Hz), often measured during the resting EC state.

The existing literature indicates that alpha reactivity to eye-opening is diminished in elderly individuals (Duffy et al., 1984; Könönen and Partanen, 1993; Wan et al., 2019) and patients with cognitive impairment, particularly Alzheimer's dementia (AD) (Franciotti et al., 2006; van der Hiele et al., 2007, 2008; Babiloni et al., 2010; Fonseca et al., 2011; Schumacher et al., 2020; Babiloni et al., 2022). Notably, neuroimaging and pharmacological studies have shed light on the neural underpinnings of altered alpha reactivity, implicating the involvement of the cholinergic system (Osipova et al., 2003; Balsters et al., 2011; Wan et al., 2019; Schumacher et al., 2020; Rea et al., 2021). Despite its clinical relevance, a comprehensive review specifically focusing on alpha reactivity in the context of cognitive aging and its underlying mechanisms remains warranted.

This article aims to review existing research on alpha reactivity across the cognitive spectrum in older adults — from healthy elders and those with subjective memory complaints (SMC) or mild cognitive impairment (MCI), to patients with AD, Lewy body dementia (LBD), and other dementias. Methodological considerations in quantifying alpha reactivity are first discussed, followed by a summary of key findings and their clinical implications. Finally, the potential neural mechanism of alpha reactivity and future research directions will be discussed. To preserve a coherent scope, this mini-review is limited to EC → EO alpha reactivity; broader constructs such as resting alpha power and the posterior dominant rhythm (PDR) are noted only for construct clarity and are not treated in depth.

## 2 Literature search

A PubMed (MEDLINE) search was conducted with the following query (last search: 2025-12-31): (EEG[tiab] OR MEG[tiab]) AND alpha[tiab] AND (reactivity[tiab] OR blocking[tiab] OR attenuation[tiab] OR desynchronization[tiab]) AND (aging[tiab] OR elderly[tiab] OR older[tiab] OR dlaementia[tiab] OR “Alzheimer disease”[tiab] OR “mild cognitive impairment”[tiab]).

Inclusion criteria were (1) articles published in a peer-reviewed journal; (2) original research presenting empirical data using EEG or MEG; (3) both EC and EO conditions explicitly measured; (4) alpha reactivity quantified as a numerical index from EC-to-EO comparison (e.g., EC/EO ratio); (5) participants ≥60 years and/or cognitively impaired. Exclusion criteria were (1) non-original articles; (2) conference abstracts; (3) younger adults or pediatric cohort only; (4) studies without numeric estimation of EC-to-EO alpha reactivity metric; (5) alpha reactivity metric not derived from a specific frequency band power (e.g., coherence or connectivity metric). This restriction was applied to align the eligibility criteria with the present operational definition of alpha reactivity (EC-to-EO change in alpha-band power).

From the initial search results (*n* = 119), titles and abstracts were screened, and full texts were assessed for eligibility based on the predefined criteria. Twenty-one original research articles met the eligibility criteria and were included in this review. To ensure coverage of mechanistic evidence, one cholinergic pharmacology study in older adults (Balsters et al., 2011) was additionally included via targeted reference screening because it reported a quantitative EC-to-EO alpha reactivity metric but was not retrieved by the MEDLINE query (it lacked reactivity-related terms in the title/abstract).

## 3 Methodological issues in alpha reactivity

### 3.1 Reactivity index

Alpha reactivity is generally quantified as the change in alpha-band power between EC and EO conditions using various metrics (Wan et al., 2019; Schumacher et al., 2020). As shown in Table 1, common indices include the ratio of EC to EO power (EC/EO) or the difference (EC–EO, often normalized by EC power). Log-transformed differences (log EC – log EO) are also sometimes used to address skewed distributions (Gasser et al., 1982; Fonseca et al., 2011). These indices are designed to quantify the degree of alpha desynchronization elicited by the transition from the EC to the EO state.

**Table 1:** Summary of EEG/MEG studies of alpha reactivity in elderly individuals.

**Authors (year)**	**Participants**	**Methods**	**Frequency**	**Spatial location**	**Reactivity index**	**Main findings**
Duffy et al. (1984)	63 healthy (age: 30–80)	EEG	IAF	Occipital (O1, O2, OZ)	EC/EO	AR negatively correlated with age.
Könönen and Partanen (1993)	54 healthy (age: 23–80)	EEG	7.57–13.92 Hz	Average (T6-O2, T5-O1)	EC/EO	AR negatively correlated with age.
Partanen et al. (1997)	17 VaD, 11 HE	EEG	7.57–13.92 Hz	Bipolar (T6-O2, T5-O1, C4-P4, C3-P3)	EC/EO	AR was smaller in VaD than HE. AR correlated positively with neuropsychological tests in VaD.
Osipova et al. (2003)	8 healthy (age: 59–80)	MEG	8–13 Hz	Frontal, Central, L/R temporal, L/R parietal, Occipital	EC/EO	AR reduced with scopolamine
Alexander et al. (2006)	79 SMC, 79 HE	EEG	8–13 Hz	All 26 electrodes	log EO/EC	No significant difference in AR between SMC vs. HE
Franciotti et al. (2006)	8 moderate AD, 7 severe AD, 7 LBD, 9 HE	MEG	7–9 Hz, 9–14 Hz	Anterior, Superior lateral, Inferior lateral, Posterior	(EC-EO)/EO	AR (9–14 Hz) was lower in AD and LBD than HE. AR (7–9 Hz) was lower in severe AD and LBD than moderate AD.
van der Hiele et al. (2007)	10 AD, 11 amnestic MCI, 12 HE	EEG	8–13 Hz	Average (all electrodes)	(EC-EO)/EC	AR was lower in AD than HE. AR correlated with cognitive performance positively.
van der Hiele et al. (2008)	8 AD, 12 MCI, 21 HE	EEG	8–13 Hz	Average (all electrodes)	(EC-EO)/EC	AR was lower in AD than HE. AR correlated with follow-up cognitive test scores.
Babiloni et al. (2010)	31 mild AD, 91 MCI, 36 HE	EEG	8–10.5 Hz, 10.5–13 Hz	Frontal, Central, Parietal, Temporal, Occipital (LORETA)	EO-EC	AR showed group difference of AD < MCI < HE. Occipital AR correlated with MMSE score positively in AD+MCI group.
Balsters et al. (2011)	14 HE (age: 55–75) in experiment 2	EEG	8–10.5 Hz, 10.5–13 Hz	Frontal, Central, Parietal, Occipital, L/R temporal	Not provided	AR (8–10.5 Hz) was smaller in donepezil administration. Significant interaction in drug ^*^ age, drug ^*^ region.
Fonseca et al. (2011)	34 AD, 30 HE	EEG	8.2–12.5 Hz	Average, L, R, Frontal, Occipital	EO/EC	AR was smaller in AD than HE (R or Occipital area).
van der Hiele et al. (2011)	11 AD, 13 MCI, 13 HE	EEG	8–13 Hz	Average (all electrodes)	(EC-EO)/EC	AR was lower in AD than HE. AR correlated with CAMCOG score positively. AR achieved good classification between AD vs. HE (AUC = 0.82)
Barry and De Blasio (2017)	20 HE (age: 60–75), 20 HY (age: 19–26)	EEG	8–13 Hz	19 electrodes	EO-EC	AR correlated with EC alpha power along 19 electrodes.
Wan et al. (2019)	19 HE (age: 60–85), 21 HY (age: 20.5 ± 2.9)	EEG/fMRI, dMRI	8–12 Hz	Occipital (O1, O2, OZ)	(EC-EO)/EC	AR correlated with reduction in the NBM to visual cortex functional connectivity upon eye opening. AR correlated with lesion in the NBM to visual cortex tracts negatively.
Chae et al. (2020)	143 MCI/SMC (70 Aβ+, 73 Aβ-)	EEG/PET	8–13 Hz	9 regions	(EC-EO)/EC	AR (left hemisphere) is lower in Aβ+ than Aβ-. AR contributed in a regression model classifying Aβ+ and Aβ- within MCI group.
Schumacher et al. (2020)	21 AD, 41 LBD, 40 HE	EEG/MRI	IAF	Occipital (O1, O2, OZ)	(EC-EO)/EC	AR and IAF decreased in order of LBD < AD < HE. AR correlated with NBM volume positively.
Fröhlich et al. (2021)	72 HE, 80 possible MCI, 17 non-amnestic MCI, 44 amnestic MCI	EEG	8–13 Hz	9 regions	log EO/EC	AR did not significantly differ between HE, possible MCI, amnestic and non-amnestic MCI.
Rea et al. (2021)	31 PD, 21 MCI, 21 HE	EEG	8–13 Hz	Occipital (O1, O2, OZ)	(EC-EO)/EC	AR was significantly lower in PD than HE. AR was positively correlated with the volume of cholinergic forebrain ROIs in PD.
Babiloni et al. (2022)	48 AD, 42 LBD, 28 HE	EEG	IAF	Frontal, Central, Parietal, Temporal, Occipital, Limbic (LORETA)	(EO-EC)/EC	AR decreased in order of LBD < AD < HC. AR correlated with global composite cognitive score. Good to moderate classification (AUC = 0.80 for LBD vs. HE, 0.76 for AD vs. HE)
Perez et al. (2022)	71 HE (34 SMC, 37 control), 75 HY (40 SMC, 35 control)	EEG	8–12 Hz	Frontal, Central, Parietal, Occipital, Temporal	log EC/EO	AR was lower in HE with SMC than HE without SMC. No group difference in healthy young groups.
Babiloni et al. (2024)	73 PDD, 35 AD, 25 HE	EEG	IAF	Frontal, Central, Parietal, Temporal, Occipital, (LORETA)	(EO-EC)/EC	AR decreased in order of PDD < AD < HC. Alpha-reactive subjects were less in PDD than HC, AD. AR correlated with MMSE. Good classification between HE vs. AD (AUC = 0.80), HE vs. PDD (AUC = 0.88).
Olǧun et al. (2024)	23 AD, 28 LBD, 15 FTD, 22 HE	EEG	8–12 Hz	Average (all electrodes)	EO/EC	AR was lower in LBD than FTD or HE. Good classification in FTD vs. LBD (AUC = 0.81).

Ach, acetylcholine; AD, Alzheimer's dementia; AR, alpha reactivity; AUC, area under receiver-operating curve; dMRI, diffusion magnetic resonance imaging; EC, eyes-close; EEG, electroencephalography; EO, eyes-open; fMRI, functional magnetic resonance imaging; FTD, fronto-temporal dementia; HE, healthy elderly; HY, healthy young; IAF, individualized alpha frequency; L, left hemisphere; LBD, lewy body dementia; MCI, mild cognitive impairment; MEG, magnetoencephalography; MRI, magnetic resonance imaging; NBM, basal nucleus of Maynert; PD, Parkinson's disease without dementia; PDD, Parkinson's disease dementia; PET, positron emission tomography; R, right hemisphere; ROI, regions of interest; SMC, subjective memory complaints.

While 13 out of 22 studies define the alpha reactivity index such that higher values indicate stronger EC-to-EO alpha desynchronization (e.g., EC/EO or EC–EO), 8 studies adopt the opposite convention (e.g., EO/EC or EO-EC), which may result in terminological inconsistency. To ensure clarity, this review consistently uses the term ‘larger reactivity' to refer to greater alpha suppression upon eye-opening; specific index definitions are reported in Table 1.

### 3.2 Frequency spectrum

The conventional alpha frequency spectrum of 8–13 Hz (Kane et al., 2017) has been widely used in alpha reactivity studies, as often detailed in Table 1. Beyond this, a few studies explore subdivisions, such as alpha1 (8–10.5 Hz) and alpha2 (10.5–13 Hz) (Babiloni et al., 2010; Balsters et al., 2011), or pre-alpha (7–9 Hz) and alpha (9–14 Hz) (Franciotti et al., 2006). These studies often find that lower-frequency alpha is more sensitive to pathological (Babiloni et al., 2010) or pharmacological effects (Balsters et al., 2011).

Beyond conventional fixed bands, individualized alpha frequency (IAF) approaches have been adopted in four studies, particularly in dementia cohorts (see Table 1). Here, IAF can be defined as an individual's dominant alpha-rhythm frequency, typically identified using the subject-specific peak (peak alpha frequency) of the EEG power spectral density under EC conditions over posterior regions. Duffy et al. (1984) identified the peak within an extended 7–14 Hz range in the EC spectrum, and then quantified alpha power/reactivity at that single peak frequency. Schumacher et al. (2020) estimated the individual alpha peak from the EC spectrum using an extended 4–14 Hz range, and quantified alpha power within a ±2 Hz window around that peak to compute reactivity. Babiloni et al. (2022, 2024) individualized frequency bands using two EC spectral landmarks: The theta–alpha transition (the local minimum between 3–8 Hz) and the background alpha peak (the maximum peak between 6–14 Hz), which were then used to derive subject-specific alpha sub-bands.

This approach is critical because elderly individuals, particularly those with AD and LBD, often exhibit slowing of the alpha rhythm in EC condition (Franciotti et al., 2006; Schumacher et al., 2020; Babiloni et al., 2022). This slowing means that a significant portion of the resting rhythm may fall outside the conventional 8–13 Hz band, which may lead to an underestimation of EC alpha power and potentially confounding the alpha reactivity measurement. Previous studies estimated the mean IAF in healthy controls (HC), AD, and LBD as 8.8 Hz, 7.4 Hz, and 6.4 Hz (Schumacher et al., 2020); or 8.9 Hz, 8.1 Hz, and 7.4 Hz, respectively (Babiloni et al., 2022).

In conclusion, while the conventional 8–13 Hz alpha band remains widely used, IAF is a more robust approach for estimating alpha reactivity in patients with dementia, particularly those with LBD and AD, who frequently exhibit posterior alpha rhythm slowing. However, IAF estimation may be unreliable or infeasible in some older or clinical recordings (e.g., flat spectra, low SNR, or ill-defined alpha peaks), which can limit its practical applicability.

### 3.3 Spatial properties

Alpha rhythms during EC rest are strongest over occipital regions, where alpha reactivity is also most prominent. As summarized in Table 1, many studies have quantified alpha reactivity either by averaging all electrodes or occipital electrodes only.

Some studies have investigated alpha reactivity across various brain regions (e.g., frontal, central, parietal, or occipital areas) by averaging power from respective electrodes. Of these, occipital and posterior regions are most consistently associated with altered alpha reactivity due to cognitive impairment or pharmacological interventions (Franciotti et al., 2006; Balsters et al., 2011; Fonseca et al., 2011). While less common, central regions have also been implicated in a study comparing elderly individuals with and without subjective memory complaints (Perez et al., 2022).

Beyond surface electrode analysis, source estimation techniques, including low-resolution electromagnetic tomography (LORETA), have been employed to quantify alpha reactivity by localizing current density in cortical regions of interest (Pascual-Marqui et al., 2002). Babiloni's group found that while significant group differences in alpha reactivity were present in occipital, parietal, and temporal regions, only occipital alpha reactivity significantly correlated with cognitive scores in MCI and AD patients (Babiloni et al., 2010). Furthermore, their work indicates that occipital alpha reactivity alone differentiated LBD from AD, even when parietal regions also showed group differences (Babiloni et al., 2022).

In conclusion, the occipital region is most consistently found to be sensitive in detecting reduced alpha reactivity related to aging or cognitive impairments. Nevertheless, averaging over the whole scalp may still provide valid information for detecting disease-related changes.

## 4 Alpha reactivity in healthy elderly individuals

Healthy aging is consistently associated with reduced EC → EO alpha suppression, indicating an age-related decline in alpha reactivity across studies. In an EEG study of healthy men (*n* = 63; age 30–80) using IAF, Duffy et al. (1984) reported a significant negative association between age and alpha reactivity (*r* = −0.434, *p* < 0.001) over occipital electrodes. In an EEG study of healthy adults (*n* = 54; age 23–80) using 7.57–13.92 Hz, Könönen and Partanen (1993) found a non-linear age-related reduction in alpha reactivity, with a steeper decline after age 60 (β = −0.01 per year for age < 60 vs.−0.04 per year for age ≥ 60). In an EEG study comparing healthy elderly (*n* = 20) and healthy young (*n* = 20) using 8–13 Hz, Barry and De Blasio (2017) demonstrated alpha reactivity as qualitatively smaller in the older group (especially in posterior regions), and reported that alpha reactivity scaled with EC alpha amplitude across electrodes (*R*^2^ = 0.7797).

While these studies converge on age-related reductions, they differ in frequency definition (IAF vs. fixed band) and spatial quantification (occipital vs. broader montages), which may contribute to variability in effect size and complicate direct cross-study comparisons. In addition, the positive association between alpha reactivity and EC alpha power (Barry and De Blasio, 2017) suggests that changes in baseline EC alpha rhythm could confound reactivity estimates, emphasizing the importance of reporting both EC power and reactivity (and, when possible, IAF) in aging cohorts.

## 5 Alpha reactivity in patients with cognitive impairment

### 5.1 Alzheimer's dementia and AD pathology

Across EEG/MEG studies, AD is consistently associated with smaller EC → EO alpha suppression than healthy controls, and this reduction has been reported even in relatively small-sample cohorts. In an MEG study of AD and LBD patients (8 moderate AD, 7 severe AD, 7 LBD, 9 HC), Franciotti et al. (2006) reported reduced 9–14 Hz alpha reactivity in AD and LBD compared with controls, and lower 7–9 Hz reactivity in severe AD and LBD compared with moderate AD. In EEG studies of AD, MCI, and HC using the conventional 8–13 Hz band, van van der Hiele et al. (2007, 2008, 2011) similarly reported blunted alpha reactivity in AD relative to HC (see Table 1 for details), supporting cross-modality convergence on an AD-related reduction in alpha reactivity. Later studies using diverse analytic choices (e.g., scalp averages or source-space estimates) have continued to report smaller alpha reactivity in AD than in HC (Babiloni et al., 2010; Fonseca et al., 2011; Schumacher et al., 2020; Babiloni et al., 2022, 2024).

Reduced alpha reactivity in AD also shows clinically relevant associations with cognitive performance and AD-related pathology. In an EEG study of AD and MCI patients (10 AD, 11 amnestic MCI, 12 HC) using 8–13 Hz, van der Hiele et al. (2007) reported that alpha reactivity was positively correlated with multiple cognitive measures (e.g., Cambridge Cognitive Examination and memory/language/executive tasks). In the follow-up study in 8 AD, 12 MCI, and 21 HC (van der Hiele et al., 2008), larger alpha reactivity at baseline was associated with better performance in language task (*r* = 0.38, *p* = 0.053) and trail making test (*r* = −0.33, *p* = 0.08) 15–25 month later, although these associations did not reach conventional statistical significance. In an EEG study of 31 mild AD, 91 MCI, and 36 HC using LORETA sources (alpha1: 8–10.5 Hz, alpha2: 10.5–13 Hz), Babiloni et al. (2010) reported a graded reduction in alpha reactivity (AD < MCI < controls: *p* < 0.001 for alpha1 and *p* < 0.05 to 0.001 for alpha2) and found that occipital alpha reactivity correlated with MMSE in the patient group (*r* = −0.24, *p* = 0.01. Please note that EO-EC was used as a reactivity index in this study).

Beyond group-level differences, several studies suggest that alpha reactivity contributes to classification or prediction models for AD-related outcomes, especially when combined with complementary spectral features. In an EEG/EMG study of 11 AD, 13 MCI, and 13 HC using 8–13 Hz, van der Hiele et al. (2011) reported good discrimination of AD vs. controls: Area under receiver-operating curve (AUC) = 0.82. In an EEG study using LORETA sources with IAF, Babiloni et al. (2022) demonstrated a moderate classification accuracy between 48 AD and 23 HC subjects using alpha reactivity (AUC = 0.76). In their another EEG study using LORETA sources with IAF, Babiloni et al. (2024) achieved a good classification accuracy in discriminating 35 AD and 25 HC subjects using alpha reactivity (AUC = 0.80). Classification performance may be further enhanced when alpha reactivity is combined with complementary spectral features. In an EEG study of 34 AD vs. 30 HC using alpha 8.2–12.5 Hz (and theta 4.9–7.8 Hz), Fonseca et al. (2011) reported smaller alpha reactivity in AD (notably over occipital/right-hemisphere channels) and showed improved AD vs. HC classification when combining alpha and theta reactivity in a logistic regression model.

In previous studies, AD was typically defined using clinical diagnostic criteria, with limited use of amyloid or other biological markers. Therefore, findings from clinically defined AD cohorts should be interpreted with caution. At the same time, biomarker-defined evidence remains limited but is emerging. Chae et al. (2020) extended this line by linking decreased alpha reactivity to PET-defined amyloid burden in non-demented older adults. In their EEG study of 70 Aβ+ and 73 Aβ- non-demented individuals using 8–13 Hz, Chae et al. (2020) reported reduced alpha reactivity in Aβ+ individuals (particularly left posterior regions) and demonstrated a predictive utility for amyloid positivity within MCI participants.

### 5.2 Lewy body dementia and other dementia

This section extends the discussion beyond AD to other dementia subtypes, including Lewy body dementia (LBD), Parkinson disease dementia (PDD), fronto-temporal dementia (FTD), and vascular dementia (VaD). Here, LBD is used as an umbrella term for dementia syndromes within the Lewy body disease spectrum (often encompassing dementia with Lewy bodies, DLB, and PDD in the clinical literature). While reduced EC → EO alpha reactivity appears to be a transdiagnostic finding across multiple dementia syndromes, accumulating evidence suggests that LBD may show particularly pronounced impairment and subtype-specific patterns.

Three studies reported that LBD patients showed typically smaller alpha reactivity compared to AD patients (Franciotti et al., 2006; Schumacher et al., 2020; Babiloni et al., 2022). In a MEG study of 8 moderate AD, 7 severe AD, 7 LBD, and 9 HC using 7–9 Hz and 9–14 Hz bands, Franciotti et al. (2006) reported reduced 9–14 Hz alpha reactivity in both AD and LBD relative to controls, and further observed reduced 7–9 Hz reactivity in severe AD and LBD relative to moderate AD. In an EEG–MRI study of 21 AD, 41 LBD, and 40 HC using IAF, Schumacher et al. (2020) reported that alpha reactivity decreased in the order of LBD, AD, and controls, supporting a graded reduction pattern (LBD < AD < controls). In an EEG study of 48 AD, 42 LBD, and 28 HC using LORETA with IAF, Babiloni et al. (2022) likewise reported that alpha reactivity decreased in the order of LBD, AD, and controls, and that occipital alpha reactivity was particularly informative for differentiating LBD from AD. Babiloni et al. (2022) also demonstrated good to moderate classification accuracy with alpha reactivity (AUC = 0.80 for LBD vs. HC, AUC = 0.76 for AD vs. HC). Collectively, these MEG/EEG studies converge on a graded reduction of alpha reactivity (LBD < AD < HC), with particularly informative effects in posterior/occipital regions.

In an EEG–MRI study of 21 AD, 41 LBD, and 40 HC using IAF, Schumacher et al. (2020) further characterized distinct subtype profiles such that AD was marked by reduced baseline alpha power in the EC condition, whereas LBD showed relatively preserved EC alpha power but a more pronounced failure of alpha desynchronization upon eye-opening. This pattern-based distinction complements magnitude-based findings and provides a mechanistic framing for why LBD may exhibit disproportionately blunted alpha reactivity. However, this subtype-specific dissociation (reduced EC alpha power in AD vs. preserved EC alpha power with impaired EO desynchronization in LBD) has been reported primarily in this single EEG–MRI cohort, and independent replication remains limited.

In an EEG study of 73 PDD, 35 AD, and 25 HC using LORETA with IAF, Babiloni et al. (2024) reported alpha reactivity decreased in the order of PDD, AD, and controls, consistent with the extension of alpha reactivity impairment across the Lewy body disease spectrum. In the same study, Babiloni et al. (2024) further reported good classification performance for PDD vs. HC (AUC 0.88) and AD vs. HC (AUC 0.80), supporting the clinical relevance of alpha reactivity in Lewy body spectrum-related dementia. Compared with LBD and AD, however, the evidence base for PDD and other non-AD dementia subtypes remains relatively limited, and future studies with independent cohorts will be important to clarify subtype-level profiles and the generalizability of diagnostic performance across analytic approaches.

Compared with AD and Lewy body disease spectrum dementia, evidence from other dementia subtypes remains limited. In an EEG study of 23 AD, 28 LBD, 15 FTD, and 22 HC using 8–12 Hz, Olǧun et al. (2024) reported that alpha reactivity was lower in LBD than in FTD or healthy controls, and further reported good discrimination between FTD and LBD (AUC 0.81), highlighting potential utility for differential diagnosis in clinically ambiguous cases. However, this finding is currently supported by a small number of cross-sectional datasets, and replication across independent FTD and LBD cohorts (with harmonized preprocessing and reactivity metrics) is needed. In an EEG study of 17 VaD and 11 HC using 7.57–13.92 Hz, Partanen et al. (1997) reported smaller alpha reactivity in VaD than controls and positive associations between alpha reactivity and neuropsychological test performance within the VaD group. However, broader replication across VaD and other non-AD/non-LBD dementias is still scarce.

### 5.3 Mild cognitive impairment and subjective memory complaints

In this section, MCI and SMC are treated as clinical syndromes, as etiological confirmation is not available in the cited studies. Across studies, MCI has often been reported to show an intermediate magnitude of alpha reactivity between AD and HC, although the statistical significance of between-group differences varies across studies.

In early studies with small samples, MCI-related group differences in alpha reactivity were reported descriptively but did not reach statistical significance. In an EEG study of 10 AD, 11 amnestic MCI, and 12 HC using 8–13 Hz, van der Hiele et al. (2007) reported that MCI patients showed alpha reactivity values intermediate between AD and HC, but between-group differences were not statistically significant. In their follow-up studies in a similar clinical cohort using the same 8–13 Hz approach (van der Hiele et al., 2008, 2011), van der Hiele et al. again described an intermediate MCI pattern, while evidence for robust separation between MCI and HC remained limited.

With larger samples, statistically significant MCI-related differences have been reported, supporting a graded pattern across the cognitive spectrum. In an EEG study of 31 mild AD, 91 MCI, and 36 HC using LORETA analysis with alpha1 (8–10.5 Hz) and alpha2 (10.5–13 Hz), Babiloni et al. (2010) reported significant group differences in alpha reactivity, with MCI showing reactivity greater than AD but less than controls (*p* < 0.05).

However, not all large cohort studies have replicated clear MCI subgroup effects. In an EEG study of 213 non-demented older adults categorized into HC (*n* = 72), possible MCI (*n* = 80), non-amnestic MCI (*n* = 17), and amnestic MCI (*n* = 44), Fröhlich et al. (2021) reported no significant differences in alpha reactivity in 8–13 Hz across these four groups. This null finding may reflect cohort characteristics (e.g., advanced mean age: 82.5 years) and/or heterogeneity in diagnostic definitions and analytic choices, which can influence sensitivity to subtle group differences in non-demented populations.

Evidence linking subjective memory complaints (SMC) to alpha reactivity is also mixed. In an EEG study of 71 older adults and 75 young adults using 8–12 Hz, Perez et al. (2022) reported significantly reduced alpha reactivity in the central region of older adults (55–74 years old) with SMC compared to those without SMC (*p* = 0.046), whereas effects were not emphasized as global scalp differences. In contrast, in an EEG study of 79 healthy elderly subjects with SMC and 79 healthy controls without MCI using 8–13 Hz, Alexander et al. (2006) reported no significant difference in alpha reactivity between SMC and controls.

Taken together, evidence for reduced alpha reactivity in MCI/SMC is suggestive but inconsistent, potentially due to heterogeneity in syndrome definitions and alpha reactivity quantification across studies. This highlights the need for larger, adequately powered cohorts with harmonized diagnostic criteria and standardized alpha reactivity quantification.

## 6 Cholinergic system in alpha reactivity

### 6.1 Pharmacological evidence for cholinergic involvement

Pharmacological studies provide causal (though still limited) evidence that cholinergic signaling modulates EC → EO alpha reactivity in older adults. Across the two available studies, cholinergic manipulation was associated with altered alpha reactivity, supporting a link between acetylcholine transmission and alpha desynchronization to eye opening.

In a MEG study of 8 healthy elderly individuals using 8–13 Hz, Osipova et al. (2003) administered scopolamine (a muscarinic acetylcholine receptor antagonist) in a double-blind, randomized, cross-over design and reported significantly diminished alpha reactivity (along with reduced theta- and beta-band reactivity). In an EEG study of 14 healthy elderly participants using alpha1 (8–10.5 Hz) and alpha2 (10.5–13 Hz) bands, Balsters et al. (2011) reported that alpha reactivity in the 8–10.5 Hz range was significantly reduced after donepezil (an acetylcholinesterase inhibitor) administration in a double-blind, crossover, placebo-controlled design.

Both studies suggest that cholinergic signaling is involved in modulating alpha reactivity to eye opening in older adults. However, these findings are derived from small samples and heterogeneous paradigms. The observation that both muscarinic antagonism and acetylcholinesterase inhibition are associated with reduced reactivity underscores that the direction and specificity of cholinergic effects on alpha reactivity remain to be clarified, particularly in larger cohorts including patients with dementia.

### 6.2 Neuroimaging studies

Several neuroimaging studies combined with EEG alpha reactivity measurement investigated the neural basis of alpha reactivity to eye opening, focusing on cholinergic basal forebrain projections including the nucleus basalis of Maynert (NBM). In a multimodal EEG–MRI study of 19 healthy older adults (age ≥ 60) using IAF, Wan et al. (2019) found that larger alpha reactivity was associated with stronger EC → EO changes in functional connectivity between the visual cortex and the NBM. Furthermore, the volume of leukoaraiosis (white matter lesions associated with aging) within the NBM-to-visual cortex projections was negatively correlated with alpha reactivity. In an anatomical MRI-EEG study of 21 AD, 41 LBD, and 40 HC using IAF, Schumacher et al. (2020) reported that NBM volume was reduced in AD and LBD and positively correlated with alpha reactivity, linking structural cholinergic degeneration to impaired EC → EO desynchronization. In a structural MRI–EEG study of 31 Parkinson's disease without dementia (PD), 21 MCI, and 21 HC using conventional 8–13 Hz band, Rea et al. (2021) reported that PD patients exhibited significantly smaller alpha reactivity compared to healthy controls (*p* = 0.04). In these patients, alpha reactivity was positively correlated with volume of posterior NBM and medial septum regions.

Taken together, these findings converge on a model in which degeneration of cholinergic basal forebrain nuclei, especially the NBM, contributes to reduced alpha reactivity across healthy aging, Parkinsonian syndromes, and dementia. However, current evidence is constrained by modest sample sizes, cross-sectional designs, and heterogeneous diagnostic groups, and it remains uncertain to what extent NBM atrophy and diminished alpha reactivity are causally linked versus reflecting parallel manifestations of broader neurodegeneration.

## 7 Future directions

EEG alpha reactivity diminishes with aging and cognitive decline, with particularly pronounced reductions observed in LBD and AD. Neuroimaging and pharmacological evidence also suggest the involvement of the cholinergic system in alpha reactivity to eye-opening. Thus, alpha reactivity can be a potential biomarker for cognitive decline reflecting cholinergic system integrity in elderly individuals. However, several questions remain, largely due to heterogeneity in reactivity metrics, frequency definitions, and cohort characterization across studies.

First, quantifying alpha reactivity in elderly populations requires careful consideration of PDR slowing. While alpha is the dominant rhythm in healthy adults during EC rest, PDR peak frequency often shifts to lower frequencies in older adults, especially those with dementia (Franciotti et al., 2006; Schumacher et al., 2020; Babiloni et al., 2022). Slowing of alpha rhythm was also associated with longitudinal cognitive decline among healthy old adults (Prichep et al., 2006). This frequency shift may attenuate EC alpha power, thereby confounding the estimation of alpha reactivity. Although IAF can mitigate this, it remains unclear whether reduced alpha reactivity and PDR slowing share common neurobiological mechanisms. Future studies should therefore report both EC alpha power and alpha reactivity (ideally with IAF-based bands) within the same analysis framework, and directly test whether reactivity and slowing provide independent vs. overlapping information for cognitive outcomes. Further investigation into the direct correlation between EEG slowing and alpha reactivity, as well as their underlying neural mechanisms, is needed.

Second, the precise contribution of the cholinergic system to alpha reactivity needs further investigation with more diverse and direct evidence. Current neuroimaging and pharmacological studies are limited in number and design. For instance, molecular imaging like positron emission tomography (PET) can assess cholinergic function in the brain of elderly individuals (Kanel et al., 2021). Pharmacological effect of donepezil (or other cholinergic agents) on alpha reactivity in dementia patients could also provide crucial causal evidence. An important open question is whether alpha reactivity can serve as a predictor of clinical responsiveness to cholinergic therapies in patients with dementia. To strengthen causal inference, future pharmacological studies should include placebo-controlled designs when feasible, and relate reactivity changes to both symptomatic outcomes and cholinergic target engagement (e.g., PET markers) within the same participants.

Third, further research is warranted to investigate the mechanisms underlying the distinct alpha reactivity patterns observed across LBD and other dementia subtypes (Schumacher et al., 2020; Olǧun et al., 2024). Replication in independent cohorts and multimodal studies incorporating EEG, MRI, and PET imaging will be critical for validating these subtype-specific alpha reactivity patterns and elucidating their underlying neural substrates. The cholinergic system including the NBM is a likely contributor, but a deeper understanding of the neurophysiological basis of EEG alterations in these dementia subtypes remains necessary. Because subtype comparisons may be sensitive to analytic choices (frequency band definition, spatial averaging vs. source analysis, and reactivity metric conventions), replication efforts should prioritize harmonized preprocessing pipelines and transparent reporting to enable cross-cohort comparability.

Finally, a longitudinal approach is crucial for investigating temporal changes and causal relationships in alpha reactivity. van der Hiele et al. (2008) provided preliminary evidence suggesting baseline alpha reactivity may predict decline in MCI; however, the finding only represented a statistical trend. Large prospective cohorts should incorporate resting-state EEG as well as cognitive assessments and neuroimaging to uncover underlying causal relationships in older populations, particularly those with progressive decline. Longitudinal designs should also account for inter-individual variability in pharmacological responsiveness, which could help identify predictive EEG biomarkers for treatment efficacy. In addition, longitudinal studies should evaluate test–retest reliability of alpha reactivity and define clinically meaningful change thresholds, since biomarker utility depends not only on group differences but also on within-individual stability over time.

## 8 Conclusion

In summary, EEG alpha reactivity emerges as a non-invasive marker that shows a consistent decline with aging and further impairment in dementia. A growing body of evidence supports its clinical relevance, particularly in patients with AD and LBD. Recent studies have demonstrated promising classification performance, including in differential diagnosis, indicating its potential for early detection and individualized therapeutic interventions. Neuroimaging and pharmacological studies implicate the central cholinergic system—particularly the NBM—in alteration of alpha reactivity in older adults, suggesting its utility as a quantifiable indicator of cholinergic integrity and function. Despite these promising findings, it is important to acknowledge that much of the current evidence is derived from small-scale, cross-sectional studies. Future work must clarify the link between EEG slowing and reactivity, probe cholinergic mechanisms with multimodal data, uncover the neural basis of subtype-specific patterns in dementia, and employ longitudinal designs. Ultimately, these efforts are essential to translate alpha reactivity from a promising research phenomenon into a validated clinical tool for managing cognitive aging.
